# Progressive right ventricular functional and structural changes in a mouse model of pulmonary arterial hypertension

**DOI:** 10.1002/phy2.184

**Published:** 2013-12-15

**Authors:** Zhijie Wang, David A. Schreier, Timothy A. Hacker, Naomi C. Chesler

**Affiliations:** 1 Department of Biomedical Engineering, University of Wisconsin – Madison, Madison, 53706, Wisconsin; 2 Department of Medicine, University of Wisconsin, Madison, 53706, Wisconsin

**Keywords:** RV dysfunction, RV overload, SUGEN, ventricular–vascular coupling

## Abstract

Right ventricle (RV) dysfunction occurs with progression of pulmonary arterial hypertension (PAH) due to persistently elevated ventricular afterload. A critical knowledge gap is the molecular mechanisms that govern the transition from RV adaptation to RV maladaptation, which leads to failure. Here, we hypothesize that the recently established mouse model of PAH, via hypoxia and SU5416 treatment (HySu), captures that transition from adaptive to maladaptive RV remodeling including impairments in RV function and decreases in the efficiency of RV interactions with the pulmonary vasculature. To test this hypothesis, we exposed C57BL6 male mice to 0 (control), 14, 21, and 28 days of HySu and then obtained synchronized RV pressure and volume measurements in vivo. With increasing HySu exposure duration, arterial afterload increased monotonically, leading to a continuous increase in RV stroke work, RV fibrosis, and RV wall stiffening (*P* < 0.05). RV contractility increased at 14 days of HySu exposure and then plateaued (*P* < 0.05). As a result, ventricular–vascular coupling efficiency tended to increase at 14 days and then decrease. Our results suggest that RV remodeling may begin to shift from adaptive to maladaptive with increasing duration of HySu exposure, which would mimic changes in RV function with PAH progression found clinically. However, for the duration of HySu exposure used here, no drop in cardiac output was found. We conclude that the establishment of a mouse model for overt RV failure due to PAH remains an important task.

## Abstract (precis)

This article describes the progressive changes in mouse right ventricle (RV) structure and function during the pulmonary arterial hypertension development. The findings may shed light on the transition from adaptive to maladaptive RV remodeling, which eventually leads to failure.

## Introduction

Pulmonary arterial hypertension (PAH) is the most severe form of pulmonary hypertension due to its rapid progression to right ventricular (RV) failure (McLaughlin et al. 2009). It is manifested as marked arterial remodeling and occlusion, mostly at the small distal arterioles, as well as mechanical stiffening of both proximal and distal pulmonary arteries (McLaughlin et al. 2009; Wang and Chesler 2011). Left untreated, the estimated median survival of PAH is 2.8 years (D'Alonzo et al. 1991; Humbert et al. 2010). Historical perspectives of PAH focus on the pulmonary vascular changes during PAH progression and thus treatment strategies often target these changes. However, even with modern therapy, the clinical outcomes of PAH are poor (Voelkel et al. 2012), which suggests that understanding the mechanisms that underlie RV dysfunction and failure may be critical to more successful treatments of the disease.

A well‐established rat model to study PAH is the combination of chronic hypoxia with antiproliferation treatment by SU5416 (i.e., HySu exposure) (Taraseviciene‐Stewart et al. 2001) (Also, see a recent review from Nicolls et al. 2012). However, unless genetic modification is employed, no known treatment creates severe or irreversible PAH in mice (Gomez‐Arroyo et al. 2012). Mice are advantageous as a species for modeling human disease because they can be more easily genetically modified and are less expensive for long‐term studies compared to rats and other larger animals. Recently, it has been reported that after 21 days of HySu exposure, C57BL6 mice develop pulmonary hypertension that recapitulates hallmarks of human PAH, especially distal arteriolar neointima formation and obliteration (Ciuclan et al. 2011). Incipient RV failure (defined by decreased cardiac output) was reported after 21 days of HySu exposure; however, the changes in RV function were not examined comprehensively. Thus, the suitability of this model for studying the molecular mechanisms of RV dysfunction and even failure remains unclear.

Our group has recently established expertise and techniques to measure hemodynamics and evaluate RV function in mice in vivo (Tabima et al. 2010). Employing these methodologies, here we investigate the hypothesis that the HySu mouse model recapitulates the transition from compensatory, adaptive RV remodeling to noncompensatory, maladaptive RV remodeling found in PAH clinically. Unlike in the Ciuclan et al.'s (2011) study, we did not observe a significant drop in cardiac output, but we did find changes indicative of RV dysfunction with PAH progression. Furthermore, our hemodynamic data are suitable for identifying a mathematical model to provide additional insights into the tissue‐level RV and pulmonary vascular changes that occur in this mouse model of PAH (Tewari et al. 2013, companion paper).

## Methods

### Animals

Male C57BL6/J mice, with a body weight of 23.6 ± 0.3 g, were obtained from Jackson Laboratory (Bar Harbor, ME). These mice were randomized into four groups (Normoxia, 14‐day HySu, 21‐day HySu, and 28‐day HySu) and then exposed to 0 (*N* = 8), 14 (*N* = 9), 21 (*N* = 9), or 28 (*N* = 8) days of normobaric hypoxia (10% oxygen) created in an environmentally controlled chamber in which nitrogen was mixed with room air (Ooi et al. 2010). The mice exposed to hypoxia were also treated with SUGEN (SU5416; Sigma, St. Louis, MO) at a dose of 20 mg/kg once weekly via intraperitoneal (i.p.) injection. The hypoxia chamber was opened for 10–20 min two to three times per week to give injections, change cages, and replenish food and water. The normoxia control group was housed in room air and treated with vehicle (CMC [0.5% (w/v) carboxymethylcellulose sodium, 0.9% (w/v) sodium chloride, 0.4% (v/v) polysorbate 80, 0.9% (v/v) benzyl alcohol in deionized water] solution) once weekly via i.p. injection for 3–8 weeks. All mice were 10–12 weeks old at the time of euthanasia. All mice were exposed to a 12‐h light‐dark cycle. The University of Wisconsin Institutional Animal Care and Use Committee approved all procedures.

### Anesthesia, ventilation, and ventricular exposure

For in vivo RV pressure–volume (PV) measurements, mice were anesthetized with urethane solution (1000–1200 mg/kg‐bw i.p.), intubated, and placed on a ventilator (Harvard Apparatus, Holliston, MA) using a tidal volume of ~225 *μ*L and respiratory rate of ~125 breaths/min. They were then placed supine on a heated pad to maintain body temperature at 38° to 39°C. A ventral midline skin incision was made from the lower mandible inferior to the xiphoid process. The thoracic cavity was entered through the sternum. The chest wall and lungs were carefully retracted to expose the RV. Hydroxyethylstarch (~24 *μ*g) (6%; 2 mg/g body weight) was injected intravenously to restore vascular volumes as reported previously (Pacher et al. 2008; Porterfield et al. 2009).

### Instrumentation and in vivo hemodynamic measurements

The left carotid artery was cannulated with a 1.2 F catheter tipped pressure transducer (Scisense, London, Ontario, Canada) and advanced into the ascending aorta to measure systemic blood pressure. Subsequently, the apex of the RV was localized and a 1.4 F admittance PV catheter (Scisense, Ithaca, NY) was introduced using a 20‐gauge needle leaving the pericardium intact. This apical puncture technique was used because the stiffness of the admittance catheter precludes a closed chest approach with catheter insertion through the jugular vein, which has been used previously to measure right ventricular pressure (only) in mice (Tabima et al. 2010). After instrumentation was established and initial RV PV measurements were obtained, the inferior vena cava was isolated and briefly occluded to obtain alterations in venous return for determination of end‐systolic and end‐diastolic pressure relations. This vena cava occlusion (VCO) was limited to a few seconds in duration to avoid reflex responses. VCO was performed at least three times. Measurements were obtained under normoxic ventilation conditions. One mouse from the 21‐day group died after instrumentation was established but before hemodynamic data could be obtained; one mouse from the 0‐day group exhibited abnormally high pulmonary pressure (computed mean pulmonary arterial pressure >35 mmHg). Therefore, these two mice were excluded from our data analysis and *N* = 8–9 per group.

The magnitude and phase of the electrical admittance as well as the RV pressure were continuously recorded at 1000 Hz and analyzed on commercially available software (Notocord Systems, Croissy Sur Seine, France). These methods are identical to those previously established by our group (Tabima et al. 2010).

After the in vivo hemodynamic measurements, animals were euthanized by exsanguinations under anesthesia and then RV free wall, left ventricle (LV) and septum tissue were harvested and weighted. RV hypertrophy was calculated by Fulton index as the weight ratio of RV and (LV + septum). Then, RV tissue was saved frozen (−80°C) for biochemical analyses. Hematocrit was obtained for each mouse immediately after euthanasia.

### Hemodynamic data analysis

The pressure and volume signals were visually checked for quality and recorded for later analysis. At least 10 consecutive cardiac cycles free of extrasystolic beats were selected and used for the analysis. Standard hemodynamic variables including heart rate (HR), RV peak systolic pressure (RVSP), total PVR (TPVR, estimated as RVSP/cardiac output), and RV function parameters such as stroke volume (SV), stroke work (SW), ejection fraction (EF), cardiac output (CO = SV × HR), chamber compliance (ΔV/ΔP), pulse pressure (PP), and effective arterial elastance (*E*_a_) were calculated. RV contractile function was quantified in three ways: as the slope of the end‐systolic pressure–volume relations (ESPVR) (*E*_es_), preload‐recruitable stroke work (PRSW), and d*P*/d*t*_max_. RV end‐diastolic indices such as d*P*/d*t*_min_, end‐diastolic volume (EDV), and relaxation factor *τ* were calculated as well. Finally, ventricular–vascular coupling efficiency (*η*) was calculated as *E*_es_/*E*_a_. Details on the calculation of the above parameters have been reported previously by our group (Tabima et al. 2010). To estimate the efficiency of each contractile myofilament, we further calculated the SW density (mmHg/beat) as SW (*μ*L mmHg^−1^ beat^−1^) per RV free wall tissue volume assuming a tissue density of 1.053 g/mL for all groups (Vinnakota and Bassingthwaighte 2004).

### Biochemical analysis

To examine the RV fibrosis during PAH, collagen content and cross‐linking were measured in frozen RVs biochemically by hydroxyproline (OHP) and pyridinoline (PYD) using adapted methods established in previous studies (Ooi et al. 2010; Wang and Chesler 2012). For each RV, random cut of the RV tissue sample was performed to allow collagen content and cross‐linking measurements from single specimen. The results are presented as *μ*g of OHP per RV tissue weight (*μ*g/mg) or nmol of PYD per RV tissue weight (nmol/mg). *N* = 4–6 per group for the OHP assay and *N* = 3–6 per group for the PYD assay.

### Statistical analysis

Statistical analysis of in vivo hemodynamics was performed using a one‐way analysis of variance (ANOVA) with Dunnett's test for exposure group (Normoxia vs. 14‐day HySu/21‐day HySu/28‐day HySu) or generalized least squares with multiple comparisons for exposure. To identify the correlation between RV collagen content and RV wall compliance, we applied the nonparametric Spearman rank correlation test with a permutation analysis using SAS version 9.2, in combination with a simple linear correlation analysis by Microsoft Excel as used previously(Ooi et al. 2010; Wang et al. 2013). Data analysis was conducted using the R software version 2.5.1 (R Foundation for Statistical Computing, Vienna, Austria). All *P*‐values were two sided and *P* < 0.05 was taken as statistically significant. All values are presented as mean ± SE.

## Results

### Progression of PAH

The success of the HySu treatment in generating severe PAH was evidenced by the measurements of RVSP (Table 1), which increased progressively with increased HySu exposure duration (*P* < 0.05). This chronic increase in RV afterload led to RV hypertrophy, which was measured by Fulton index (Table 1; *P* < 0.05). In addition, mice in the HySu exposure groups had increased hematocrit (Table 1; *P* < 0.05). We did not observe significant changes in systemic pressures or LV mass (Table 1) between the experimental groups.

**Table 1.** Changes in RV with 14‐, 21‐, and 28‐HySu compared to Normoxia

Group	Normoxia	14‐HySu	21‐HySu	28‐HySu
RVSP	26 ± 1	37 ± 2*	41 ± 2*	45 ± 1*
RVEDP	0.8 ± 0.1	1.2 ± 0.1	1.4 ± 0.1*	1.4 ± 0.2*
LV+S	78.3 ± 2.2	77.8 ± 4.3	79.5 ± 2.6	78.9 ± 4.5
BW	25.3 ± 0.6	23.2 ± 0.4	23.2 ± 0.9	24.1 ± 0.6
Fulton index	0.26 ± 0.01	0.40 ± 0.02*	0.43 ± 0.01*	0.44 ± 0.03*
Hct	46 ± 1.1	75 ± 2.2*	74 ± 1.2*	61 ± 0.4*
RV compliance	0.53 ± 0.03	0.36 ± 0.04*	0.36 ± 0.03*	0.30 ± 0.01*
*τ*	5.7 ± 0.5	3.1 ± 0.6*	4.9 ± 0.4	5.0 ± 0.3
EDV	23.8 ± 1.0	26.4 ± 3.1	28.9 ± 2.2	29.9 ± 1.5
ESV	9.8 ± 0.6	12.4 ± 2.6	14.8 ± 1.9	16.5 ± 1.3
SV	14.7 ± 1.0	14.9 ± 1.4	14.2 ± 1.0	13.4 ± 0.7
HR	552 ± 16	597 ± 17	591 ± 19	597 ± 17
Aortic pressure	53 ± 5	55 ± 3	59 ± 1	55 ± 1

*N* = 8–9 per group. RVSP (mmHg), RV end‐diastolic pressure (RVEDP, mmHg), LV + Septum mass (LV+S, mg), body weight (BW, g), Fulton index (mg/mg), hematocrit (Hct,%), RV compliance (*μ*L/mmHg), relaxation factor *τ* (msec), end‐diastolic volume (EDV, *μ*L), end‐systolic volume (ESV, *μ*L), stroke volume (SV, *μ*L), heart rate (HR, beats/min), aortic pressure (mmHg).

* *P* < 0.05 versus Normoxia.

### Changes in RV function

**Figure 1.**
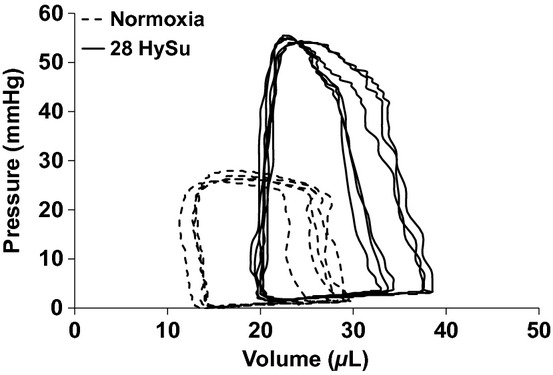
Representative in vivo RV pressure–volume loops from normoxia (dashed line) and 28‐HySu mice (solid line), respectively.

**Figure 2.**
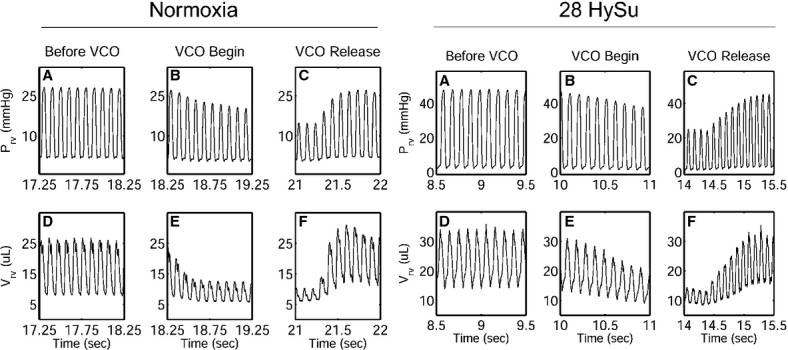
Representative in vivo RV pressure and volume waveforms with the inferior vena cava occlusion (VCO) from normoxia (left) and 28‐HySu (right) mice, respectively.

Representative RV PV loops for the normoxia control and the advanced PAH (28‐day HySu) groups are presented in Figure 1. In addition to the increase in mean and peak systolic RV pressure with PAH, both EDV and end‐systolic volume (ESV) shifted to the right and there was a noticeable but nonsignificant decrease in SV. Moreover, we found that after the VCO, it took more heart beats (cardiac cycles) for the 28‐day HySu RVs to recover compared to the normoxia control (Fig. 2), which may indicate an impaired RV response with severe PAH.

**Figure 3.**
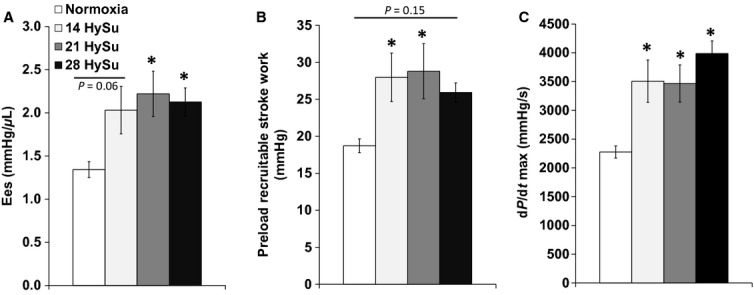
Changes in RV contractility parameters (*E*_es_, preload‐recruitable stroke work and d*P*/d*t*_max_) during the progression of PAH. *N* = 8–9 per group. *P < 0.05 versus normoxia.

We quantified RV contractile function by *E*_es_, PRSW, and d*P*/d*t*_max_. All three parameters showed the same trends of changes from an early (14‐HySu) to later (28‐HySu) stage of PAH: RV contractility tended to increase after 14 days of HySu exposure compared with control and then plateaued in the 21‐day and 28‐day HySu groups (Fig. 3).

**Figure 4.**
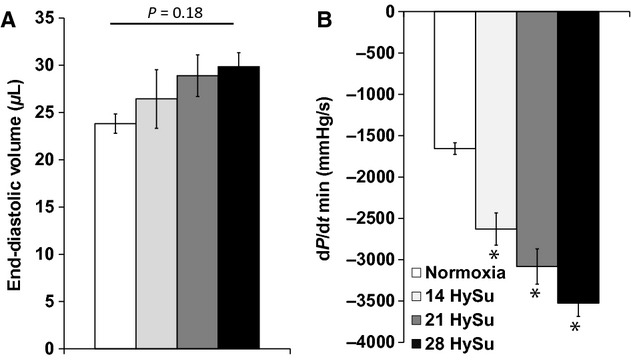
Changes in RV diastolic function parameters (end‐diastolic volume and d*P*/d*t*_min_) during the progression of PAH. *N* = 8–9 per group. *P < 0.05 versus normoxia.

To assess RV diastolic function, we measured RV chamber compliance, EDV, d*P*/d*t*_min_, relaxation factor *τ*, and RV end‐diastolic pressure (RVEDP). As shown in Table 1, we observed a progressive and significant decrease in RV chamber compliance from early PAH to later PAH (*P* < 0.05). RV EDV tended to increase with PAH (Fig. 4A). d*P*/d*t*_min_ was markedly reduced with PAH (*P* < 0.05) and continued decreasing with increasing HySu exposure duration (Fig. 4B) (*P* < 0.05). There was a significant decrease in *τ*, a preload independent measure of isovolumic relaxation, with early PAH but *τ* returned toward control levels in the 21‐HySu and 28‐HySu groups (*P* < 0.05, Table 1). We also observed a small but significant increase in RVEDP in the later PAH groups (*P* < 0.05, Table 1).

**Figure 5.**
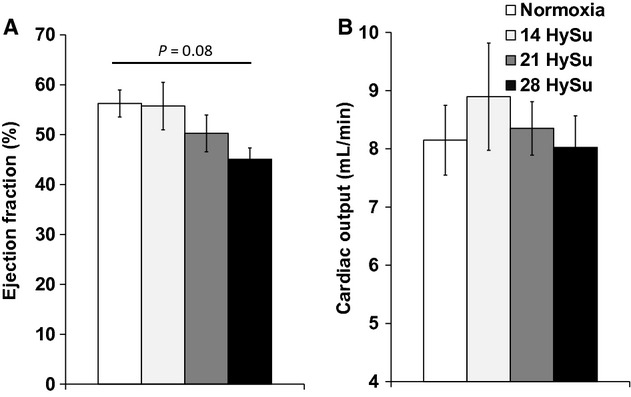
Changes in RV ejection fraction and cardiac output during the progression of PAH. *N* = 8–9 per group. *P < 0.05 versus normoxia.

We also measured the functional parameters that are typically used to evaluate heart dysfunction: EF and CO (Voelkel et al. 2006). With the progression of PAH, EF tended to decrease progressively but the changes did not reach statistical significance (Fig. 5A, *P* = 0.08). We did not observe significant changes in CO with PAH progression (Fig. 5B) and we did not observe differences in HR between the groups (Table 1).

**Figure 6.**
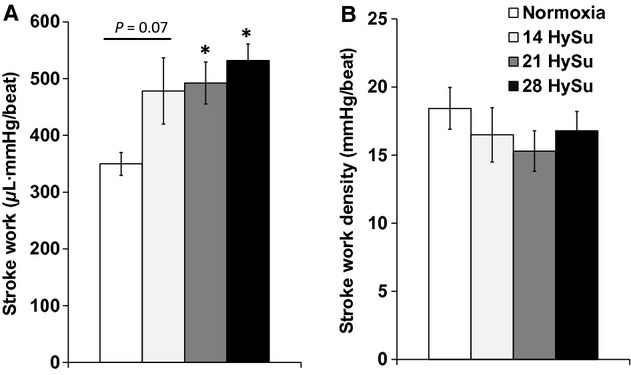
Changes in RV stroke work and stroke work density during the progression of PAH. *N* = 8–9 per group. *P < 0.05 versus normoxia.

Finally, we measured RV SW, which is the total work of the RV required to overcome the arterial afterload and maintain pulmonary flow per cardiac cycle. We found a continuous increase in SW that reached statistical significance in the 21‐HySu and 28‐HySu groups (*P* < 0.05) (Fig. 6A). Moreover, because SW density has been related to myofilament contractility and the spatial distribution of ventricular depolarization (Kerckhoffs et al. 2003), it is a useful parameter for quantifying RV function. By normalizing the RV SW to RV free wall tissue volume, we calculated RV SW density and found that it did not change with PAH progression (Fig 6B).

### Changes in the pulmonary vasculature and ventricular–vascular coupling

**Figure 7.**
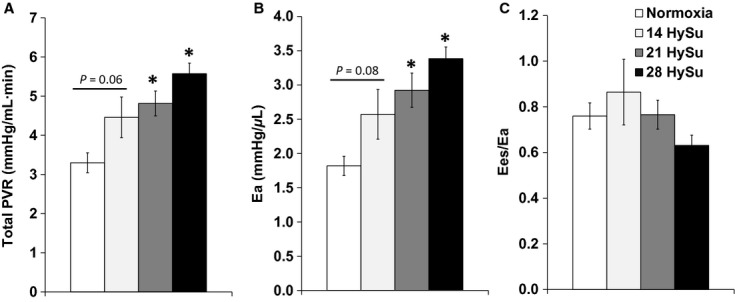
Changes in RV afterload parameters (total PVR and *E*_a_) and ventricular–vascular coupling efficiency (*E*_es _/*E*_a_) during the progression of PAH. *N* = 8–9 per group. *P < 0.05 versus normoxia.

Pulmonary vascular remodeling, especially in the small pulmonary arterioles, was evident by increased TPVR and *E*_a_. With PAH progression, TPVR and *E*_a_ increased significantly with HySu exposure (Fig. 7A and B, *P* < 0.05). Because of the continuous increase in the RV afterload up to 28 days of HySu and the plateau of elevated RV contractility after 14 days of HySu exposure, the ventricular–vascular coupling efficiency *η* tended to increase in the 14‐HySu group and then tended to decline (Fig. 7C). However, these changes in *η* did not reach significance.

### Changes in RV collagen and cross‐linking

**Figure 8.**
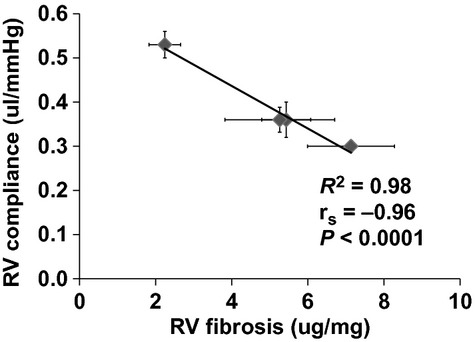
Group correlation between RV wall compliance and RV fibrosis (collagen content per unit RV free wall mass). *N* = 4–9 per group.

To understand the mechanisms for RV changes such as decreased RV wall compliance and diastolic function, we examined changes in collagen content and cross‐linking in the RV in response to HySu exposure (Table 2). RV fibrosis was evident in all HySu groups. We observed a significant increase in total collagen amount (*P* < 0.05) as well as a trend of decrease in collagen cross‐linking with PAH progression. The increase in collagen content was further augmented in 28‐HySu group compared to the 14‐ and 21‐HySu groups (*P* < 0.05). The total amount of cross‐linking tended to decrease in early PAH and then rise toward control values in later PAH. The simultaneous increase in total amount of collagen and decrease in cross‐linking led to a decrease in the density of cross‐linking, calculated as the ratio of collagen cross‐linking to content, and the reduced cross‐linking density remained similar among the three HySu groups (*P* < 0.05; Table 2). We further examined the relationship between RV collagen content and wall compliance and observed a strong correlation (*R*^2^ = 0.98, *r*_s_ = −0.96, *P* < 0.0001) (Fig. 8).

**Table 2.** Changes in RV collagen content and cross‐linking with 14‐, 21‐, and 28‐HySu compared to Normoxia

Group	Normoxia	14‐HySu	21‐HySu	28‐HySu
OHP	2.2 ± 0.2	5.4 ± 0.3*	5.3 ± 0.6*	7.1 ± 0.5*
PYD	0.20 ± 0.05	0.10 ± 0.02	0.08 ± 0.01*	0.16 ± 0.04
PYD/OHP	0.10 ± 0.03	0.02 ± 0.00*	0.02 ± 0.00*	0.02 ± 0.01*

*N* = 3–6 per group. Collagen content measured via OHP (*μ*g/mg), cross‐linking via PYD (nmol/mg), and cross‐link density via PYD/OHP (nmol/*μ*g).

* *P* < 0.05 versus Normoxia.

## Discussion

In this study, we examined the RV functional changes during PAH progression in a recently established mouse model of severe PAH that was previously reported to lead to incipient RV failure. With up to 28 days of HySu exposure, we found increased arterial afterload, RV wall stiffening, increased RV SW, and increased RV contractility. RV fibrosis was evident in both early and later PAH and RV collagen content was negatively correlated with the RV wall compliance. We also observed a trend of increase in ventricular–vascular coupling efficiency with early PAH (14‐day HySu) and then decrease with later PAH (28‐day HySu) at which point the PAH was more severe (i.e., TPVR and RVSP were greater). These results suggest that RV function may begin to transition from adaptive to maladaptive with persistent RV overload between 14 and 28 days of HySu exposure. However, we did not observe significant changes in cardiac output or EF.

The HySu treatment has been used in rats and mice to induce marked pulmonary vascular remodeling and occlusion in the small arteries, which do not occur with hypoxia exposure alone. Therefore, it is well accepted that this model generates severe pulmonary hypertension, in contrast to the hypoxia‐alone model that only generates at most moderate pulmonary hypertension. However, most studies have focused on the pathology of occlusive lesions in the pulmonary arteries, and the changes in RV function have been largely neglected. To date, the only study with extensive examination of RV remodeling in response to HySu exposure is by Bogaard et al. (2009b). In this study in rats, RV failure, defined by a drop in cardiac output that preceded increased mortality, was found to be associated with myocardial apoptosis, fibrosis, decreased RV capillary density, and a failing antioxidant defense. Because a pulmonary artery banding model that generates a comparable increase in RVSP to the HySu model did not generate RV failure (by these same metrics), Bogaard et al. questioned the common concept that RV failure is due to pressure overload. While intriguing, these studies lack thorough mechanical measurements of RV function and the interaction between the RV and pulmonary vascular bed, which makes it difficult to evaluate the performance of the right heart as a pump in the context of pressure overload.

Inspired by these prior studies, we sought to identify the changes in RV function that occur with the recently established mouse HySu model, which was reported to show signs of RV failure after 3 weeks of HySu exposure including a significant drop in CO (Ciuclan et al. 2011). Because the 3‐week exposure was reported to result in only “incipient” RV failure, we extended the HySu exposure to 4 weeks. By measuring RV function at different stages of PAH progression, we hoped to capture the progression of RV remodeling from early adaptation to maladaptation and failure, mimicking the transition from adaptive to maladaptive RV remodeling found clinically. While we did not observe a drop in CO with 28 days of HySu exposure or a significant decrease in the efficiency of interactions between the RV and pulmonary vasculature, we did observe significant changes in pulmonary vascular and RV structure and function.

With HySu exposure up to 28 days, we observed continuously increasing pulmonary arterial afterload as indicated by TPVR and *E*_a_ (Fig. 7). This suggests progressive pulmonary arterial occlusion (narrowing) with HySu exposure duration. We did not examine the histological changes in these lungs, but previous studies have reported marked distal lumen occlusion in the rodent HySu model (Taraseviciene‐Stewart et al. 2001; Abe et al. 2010; Ciuclan et al. 2011). There was also a continuous increase in RVSP (Table 1). These results suggest increasing severity of PAH as HySu exposure duration increases.

Our in vivo measurements revealed that RV function declined with the progression of PAH. We observed an increase in RV contractility in early PAH, but the increase plateaued in later PAH, up to 28 days of HySu exposure (Fig. 3), suggesting the RV managed to meet the increased demands of afterload as PAH started but then reached its maximal capacity as vascular changes worsened. The change in diastolic function was unclear. The decrease in d*P*/d*t*_min_ (more negative values) suggests an improved diastolic function, but the trends in relaxation factor (*τ*) and EDV seem to suggest the opposite. *τ* was reduced with early PAH (14‐HySu, *P* < 0.05) but the decrease was absent at later PAH. This suggests that diastolic function increased to accommodate the increasing RV afterload in early PAH but then such improvement disappeared with more progressive PAH. RVEDP increased slightly but significantly in 21‐HySu and 28‐HySu groups (Table 1). A higher RVEDP could suggest larger preload; but because we also observed RV fibrosis and decreased wall compliance, we speculate that the small increase in RVEDP is mainly a consequence of stiffer RV wall. EDV tended to increase (Fig. 4) and RV EF tended to decrease in the 28‐day HySu group (Fig. 5). Because increased EDV is a strong predictor of mortality in PAH (van Wolferen et al. 2007) and because RV EF is also closely related to the RV dilatation and has been recognized as a strong predictor of mortality in PAH (Kawut et al. 2005; van de Veerdonk et al. 2011), these trends likely indicate that RV remodeling has become maladaptive. Another sign of RV dysfunction at 28 days of HySu exposure lies in *E*_es_/*E*_a_, an index of hemodynamic coupling or the efficiency of the ventricular–vascular interactions. Interestingly, we observed a trend of increase in *E*_es_/*E*_a_ with early PAH (14‐HySu) and a possible decreasing trend with later PAH (28‐HySu) (Fig. 7). This suggests that in an early or mild stage of the disease, the RV adapts to preserve efficiency; but as disease progresses and becomes more severe, neither remodeling nor increased contractility allows the RV to meet the increasing demands. Subsequently, hemodynamic coupling efficiency decreases, as observed in patients with PAH previously (Kuehne et al. 2004; Gupta et al. 2011; Sanz et al. 2012). The lower *E*_es_/*E*_a_ may be indicative of maladaptive RV remodeling, which awaits further investigation. We speculate that RV remodeling becomes dysfunctional at 28 days of HySu and, if extended to an even longer HySu exposure time (i.e. 8~12 week), RV failure would occur.

We also examined biological changes in the RV tissues and found significant hypertrophy and fibrosis. RV hypertrophy occurred with early PAH but like contractility, did not increase much with later PAH (Table 1). In contrast, the collagen accumulation in RV continued to increase as PAH progressed, and we found a strong, negative correlation between collagen content and RV wall compliance (Table 2, Fig. 8). Similar to the LV, RV fibrosis is a hallmark of dysfunctional or failing RV (Bogaard et al. 2009a,b). It is known that the predominant matrix scaffold in the heart is collagen, which surrounds, supports, and interconnects the myocytes, myofibrils, and muscles to maintain ventricular shape and size and contributes to tissue stiffness (Janicki et al. 2006). Changes in collagen have been found to affect the myocardial systolic and diastolic functions in the pressure‐overloaded LV (Weber et al. 1988; Weber 1989; Baicu et al. 2003). For example, Lopez et al. recently showed that that increased collagen cross‐linking is associated with increased filling pressure, increased chamber stiffness, and decreased EF in LVs with chronic stage C heart failure (Lopez et al. 2012). In PAH, RV fibrosis has been correlated with elevated mean pulmonary arterial pressure and PVR (Sanz et al. 2007; Shehata et al. 2011), as well as RV hypertrophy (Shehata et al. 2011). How the collagen network affects myocyte function and eventually the macroscopic function of the RV may be critical to the mechanisms of RV failure.

In our study, we did not observe a significant drop in CO in the HySu groups, which does not agree with the prior findings of Ciuclan et al. (2011). However, Ciuclan et al. measured CO echocardiographically in the aorta, whereas we measured CO invasively in the RV. These different technical approaches may greatly affect the measurement. Furthermore, we used a different control group than Ciuclan. A drop in CO was found by Ciuclan when compared to a SUGEN‐treated control group, not a vehicle‐treated control group as we did. Another discrepancy is that the RVSP did not increase as much as previously reported (~50 mmHg in Ciuclan et al.) even after 28‐day HySu exposure, which may be related to the maintenance of CO. All procedures and treatments for the HySu model were identical between our study and the prior study, except for two aspects: (Abe et al. 2010) we used i.p. injection instead of s.c. and (Baicu et al. 2003) our mice were older (8–10 weeks old) than in the prior study (~6 weeks old) at the beginning of the HySu exposure. The ways in which these differences may affect the cardiopulmonary responses to HySu are unknown. For example, with regard to the first point, with i.p. injection the drug enters the systemic circulation via the hepatic portal system, which may induce some portal hypertension via endothelial damage. Moreover, although both the previous and current studies use C57BL6 strains, some carry a mutation in nicotinamide nucleotide transhydrogenase (NNT), a mitochondrial protein, that influences mitochondrial respiration (Ripoll et al. 2012), which would potentially affect the outcomes of the HySu exposure.

In our hands, up to 28 days of HySu exposure does not decrease the SV, which is maintained by increased EDV. Thus, at the cellular level, the Frank–Starling mechanism should be preserved. However, our data are based on whole chamber function and details of the RV myocyte length–tension relationships, which may deteriorate with PAH progression, were not directly measured. We estimated the efficiency of the myofilaments by SW density and did not find difference between the HySu groups and the control group. In the calculation of SW density, we assume constant tissue density for all experimental groups. Future examination on tissue density changes in a hypertrophied or fibrotic RV may be useful. Because we used the RV tissues for collagen quantification, we were not able to quantify RV wall thickness or myofilament density to confirm the structural changes, which can be examined in a future study. To gain further insight into potential changes in RV free wall mechanics as well as pulmonary vascular changes both such as stiffening and narrowing, Tewari et al. (2013, companion paper) fit a mathematical model of realistic ventricular mechanics coupled with a simple model of the pulmonary and systemic vascular systems to these data. These model results provide additional insights into the tissue and cellular level changes in RV and pulmonary vascular function during the progression of severe PAH and support the suggestion that the 28‐day time point may represent a transition from RV adaptive remodeling to RV maladaptive remodeling that precedes overt RV failure.

While “RV dysfunction/failure” is becoming a new research area in pulmonary hypertension (PH), its definition, especially in terms of hemodynamic measurements, remains unclear. In the literature, impaired compliance, worsened contractile function, and uncoupled ventricular–vascular efficiency have all been used as indicator of RV dysfunction or failure. Clinically, the definition of RV failure is also challenging with limited acceptance of echocardiographic measures of RV EF and limited PV loop data in this domain. Moreover, parameters like CO or EF do not necessarily change in severe patients with dysfunctional or failed hearts (Guazzi et al. 2011; Rain et al. 2013). In our study, hematocrit (Hct) increased (Table 1) in all HySu groups, which would increase blood viscosity and both systemic and pulmonary vascular resistance (Schreier et al. 2013). Our data did not allow us to distinguish the effects of increased resistance (both systemic and pulmonary) and increased oxygen carrying capacity of blood on the maintenance of CO. Since increases in Hct occur clinically (Persson et al. 1991), and these may affect CO, this is another reason maintenance of CO may not be the best measure of RV function in PH progression. Therefore, a better understanding of the key hemodynamic parameters that may indicate the transition from adaptive to maladaptive remodeling is needed. Investigations such as these, combined with other factors such as the metabolic changes that indicate the shift from compensated (adaptive) to decompensated (maladaptive) RV hypertrophy (Sutendra et al. 2013), may eventually assist in the prognosis and treatment of PAH.

## Conclusion

In summary, our results demonstrate RV functional changes with PAH development in a mouse model. With up to 28 days of HySu exposure, we found continuously increased arterial afterload and increased RV contractility that plateaued with later PAH (>14‐day HySu). RV fibrosis and hypertrophy were evident in all stages of PAH and RV collagen content was negatively correlated with the RV wall compliance. Our results suggest that RV remodeling may begin to transition from adaptive to maladaptive with persistent RV overload, which would mimic changes in RV function with PAH progression found clinically. To investigate the critical transition to RV failure, younger mice, longer exposure to HySu or a different model may be required.

## Acknowledgments

We thank Guoqing Song for surgical procedures and Jens C. Eickhoff for statistical analysis.

## Conflict of Interest

None declared.
